# Young children contribute to nature stewardship

**DOI:** 10.3389/fpsyg.2022.945797

**Published:** 2022-09-01

**Authors:** Elena Dominguez Contreras, Marianne E. Krasny

**Affiliations:** Department of Natural Resources and the Environment, Cornell University, Ithaca, NY, United States

**Keywords:** young children’s nature stewardship, early childhood environmental education, children as active citizens, children’s contribution, early childhood education for sustainability

## Abstract

Research on young children in environmental education (EE) has focused on unstructured play in, or experiencing, nature. Little attention has been paid to young children’s stewardship efforts, or to the relation of such efforts to young children’s learning and capacity to contribute to their communities and local nature. This perspectives paper draws on the first author’s experience guiding pre-k and kindergarten children (4–6 years old) in outdoor educational projects in Santo Domingo (SD), Dominican Republic, in which the children produced a park guide and a short film. In addition to becoming resources for the local community, these products are an example of children’s civic contributions. In “return on investment” language, guiding young children in outdoor experiences and reflecting on the experience represent the investment and the park guide and other products, and importantly, children’s recognition of their ability to make contributions to their community, represent the return on investment. Based on our observations that young children can make significant contributions to their communities when given the opportunity, this perspectives paper argues for a research agenda and investment in opportunities for young children to contribute to their socio-ecological communities. To support our perspective, we first review and critique the prevailing and emerging paradigms of early childhood EE, following which we briefly describe the Santo Domingo (SD) project, and close by integrating past work with the first author’s experience to argue for the importance of including young children in stewardship efforts.

## Introduction

Young children are generally viewed as “actors and creators of the future” (Heggen et al., 2019, p. 387, 2019). But what if, instead of environmental education (EE^1^) programs viewing young children as *future* adult stewards, young children engaged in stewardship, i.e., community participation to enhance circumstances for participants, the greater community of life, and future generations of both humans and nature (Elliott and Davis, 2009). Recognizing that some scholars will object to what may be seen as inappropriate pedagogies for early childhood, we argue that when adults provide age-appropriate affordances, young children are capable of contributing to nature restoration and improving their communities, and themselves benefit from such engagement. To support our perspective, we first review and critique the literature on early childhood EE approaches, and then offer a short description of programs in which young children engaged in stewardship in SD, Dominican Republic.

## Literature review

Below we review four trends in early childhood EE: Nature play, post-humanism, early childhood education for sustainability (ECEfS), and positive youth development (PYD).

### Nature-play as environmental education

EE has often neglected young children’s capacity to contribute to their local socio-ecological communities. Traditionally, EE has emphasized children as future environmental stewards and has assumed that children’s outdoor play and joyful time in nature leads to connection to or love for nature (Chawla, 1998, 1999; Hägglund and Samuelsson, 2009; Rice and Torquati, 2013; McClain and Vandermaas-Peeler, 2016) and that these dispositions will encourage children to become adults who are capable of nurturing and taking care of nature (Chawla, 1998, 1999, 2009; Wells and Lekies, 2006; McClain and Vandermaas-Peeler, 2016; Hoover, 2021). However, there is limited evidence that outdoor play and connection to nature as a child leads to adult nature stewardship (Gill, 2014; Rosa and Collado, 2019).

Research has demonstrated the social, cognitive, and health benefits of young children’s unstructured play in nature (Louv, 2008; Ardoin and Bowers, 2020). Moreover, Ernst and Burcak (2019) conducted four pilot studies of nature-based preschools where children had weekly time for play and exploration in nature. They found that play in nature promoted curiosity, creative thinking, executive functioning, and resilience, which are key problem-solving skills for humans to contribute to a sustainable future.

### Post-humanist environmental education

More recently, EE has made use of post-humanist theories and common-world pedagogies to challenge the dichotomy between human/nature and nature/culture, and contest notions that suggest (a) humans are apart from nature and (b) humans’ role is to protect nature. These scholars argue that children are nature and encourage a relational approach or kinship-making with the non-human world (Taylor, 2017; Cutter-Mackenzie-Knowles et al., 2020). Post-humanist and common world pedagogies scholars argue that stewardship pedagogies do not offer the necessary transformation to counteract the effects of the Anthropocene and to transgress the narratives that shape today’s world, and thus, they reinforce human-centric perspectives in EE (Taylor, 2017; Cutter-Mackenzie-Knowles et al., 2020). For them, educational approaches must radically change human thinking to understand that agency is a shared trait across humans, non-human species, and objects. For example, Stevenson et al. (2020) explains that non-human nature’s materiality interacts with human agency, by delineating what “humans learn about/in/for nature” (p. 1417). Thus, non-human nature is not an inactive entity for children’s knowledge and experiences, and humans should not assume the role of nature steward or conserver.

### Early childhood education for sustainability

In contrast to Post humanist approaches, Education for Sustainability (ESD) understands humans as “agents of change” (Elliott and Davis, 2009, p. 67) and focuses on the process of learning to act in a “sustainable way” (Christie and Higgins, 2012, p. 7). It is inclusive of groups that have been considered of minor importance, such as children, future generations, and non-human nature. According to Ernst and Burcak (2019), ECEfS seeks to promote children’s critical thinking and problem-solving, and children becoming “agents of change for sustainability” (p. 2) through decision-making and taking actions on local sustainability issues. Hedefalk et al. (2015) define ECEfS as an educational approach that integrates knowledge about how ecosystems function, direct experience in nature, and authentic participation in solving environmental issues; it also emphasizes the interconnected dimensions of sustainability—economic, social, and environmental.

### Positive youth development

The PYD literature is consistent with ECEfS notions of children as agents of change. Nature stewardship at an early age could promote in children a positive developmental path, with similar outcomes to PYD programs for teen-aged youth such as engaging youth in community gardening and other means of contribution to one’s community (Delia and Krasny, 2018). Lerner et al. (2005) proposed the Six C’s framework for PYD: “competence, confidence, connection, character, caring, and contribution to the community and civil society” (p. 23). The sixth C (“contribution”) refers to youth engagement in community service, local decision-making, and other activities where youth actively create positive change in their community. Several studies have linked youth contribution to outcomes for youth, including wellbeing and eco-literacy (Eccles and Gootman, 2002); ecological place meaning (Kudryavtsev et al., 2012); place-identity (Armstrong, 2022); social connections, sense of belonging and leadership (Delia and Krasny, 2018); civic skills (Russ and Gaus, 2021); academic attainment (Volk and Cheak, 2003); and connection to nature (Schusler et al., 2009).

When investments are made in adapting PYD programs for younger children, participants may experience the social and cognitive benefits that have been demonstrated for adults and youth who participate in community-based environmental stewardship or nature-restoration activities (Delia and Krasny, 2018; Russ and Gaus, 2021; Armstrong, 2022). For example, Schusler et al. (2009) found that educators who guide youth participatory stewardship and related participatory programs observe in youth increased affection for nature, recognition of social justice issues, self-esteem, self-efficacy, and citizenship skills. We contend it would be worth exploring similar participatory stewardship programs with young children.

## Young children can be nature stewards

We now turn to our perspective arguing for the importance of young children as nature stewards. In so doing, we present several arguments for children as stewards while integrating our perspective into critiques of the existing literature.

According to Serriere (2019), civic engagement at an early age occurs when children participate in improving their local context, and this participation becomes the foundation for a “lifetime of civic engagement and empowerment” (p. 384). By age four, children are capable of recognizing feelings, dispositions, abilities, and actions among their peers and adults in familiar settings, which are key social skills that enable them to cooperate with others (Flekkøy and Kaufman, 1997; Mar et al., 2010). Furthermore, preschoolers are able to use information from intentional observation and involvement to learn cause-effect relations (Kushnir et al., 2008). According to socio-cultural approaches to learning, by age five children develop through their dynamic and growing participation in the socio-cultural activities of their communities (Rogoff, 2003). In sum, young children have the capacity to participate in civic engagement activities, including stewardship, and this participation could facilitate healthy development.

In fact, early childhood is a critical time to engage in stewardship. Early childhood is the ontogenetic stage where humans learn to interact with others in their socio-cultural context and to create “humanlike social and cultural activities” (Tomasello et al., 2005, p. 676). Further, because humans learn the foundational knowledge, skills, behaviors, and values that will accompany them through life during childhood (Young and Mundial, 1996; Samuelsson, 2011), and because in an era of environmental crises learning positive ways to relate to nature should be considered a basic skill (Ärlemalm-Hagseér, 2013; Cutter-Mackenzie et al., 2014; Buil et al., 2019; Ernst and Burcak, 2019), early childhood is an ideal period for humans to learn to use their body, mind, and emotions to connect to the larger community of life through stewardship. By doing so, children can become embedded in a culture of nature caring and restoration instead of nature extraction, ethically and empathetically connect to and familiarize themselves with nature, understand the interdependency between humans and nature, and advance their social, cognitive, and wellbeing capacities, while contributing to the flourishing of the natural world.

In arguing that the early years are a decisive period for learning about and creating social and cultural practices aimed at restoring and regenerating nature, we recognize that nature play and post-humanist EE do not address children’s contribution. Current guidelines for early childhood EE focus on free playtime in nature rather than on young children responding to the environmental challenges of their communities (Ärlemalm-Hagseér, 2013; Cincera et al., 2017; Ardoin and Bowers, 2020), thus positioning children as passive agents and removing them from opportunities for civic engagement to help resolve environmental crises. Ernst and Burcak (2019) argue that cognitive skills promoted in nature-based preschools are key to solving future sustainability issues. However, scholars have challenged the assumption that young children playing in or experiencing nature will lead to stewardship and have promoted children’s direct participation in addressing environmental problems (Elliott and Davis, 2009; Davis, 2010; Blanchard and Buchanan, 2011; Cutter-Mackenzie et al., 2014; Davis and Elliott, 2014; Gill, 2014) and in practicing civic environmental skills (Ärlemalm-Hagseér, 2013). Yet, the nature-play to nature-stewardship paradigm has prevailed in EE (Ardoin and Bowers, 2020).

In addition, as post-humanist EE gains in popularity and continues to promote non-stewardship pedagogies, its proponents will need to examine questions such as children’s adaptive response to current ecological threats, and the impact of children’s actions on earth systems. In our view, stewardship and relational values can find common ground. Children should learn about and adopt ecocentric values and relational approaches to relate to non-human nature, which guide restorative and regenerating practices (stewardship) of non-human nature.

Further, post-humanist EE ignores the uniqueness of humans’ socio-cognition (Tomasello, 2019; Laland and Seed, 2021), which evolved in reaction to ecological threats that obliged humans to cooperate to gather food and protect their possessions from other groups. Human distinctive socio-cognitive skills emerge from cooperating and exchanging information and ideas while engaging in socio-cultural endeavors with other humans (Tomasello, 2019). Children inherit the sociocultural context (e.g., symbols, institutions) and their unique capacities to fully mature would be hindered without this context (Tomasello, 2019). Unfortunately, children also receive socio-cultural practices that deplete the Earth. To counteract these practices, children must participate in socio-cultural practices where they learn to be and become citizens who regenerate and positively transform their socio-ecological system. In short, we consider children *taking action* essential.

Having challenged notions about children as *future* nature stewards and non-stewardship pedagogies and having introduced our perspective about young children as social actors and agents of change, we next turn to examples of children contributing to their community.

## Young children’s contributions in Santo Domingo

The Park Guide project, conducted by kindergarteners (4–6-year-olds) and facilitated by the first author in SD, Dominican Republic, provides an example of children’s participatory stewardship. Children participating in the 9-month project explored, played in, and researched six urban parks, and then designed, wrote, and published “*Guía de Parques Divertidos*” (*Fun Park Guide*), a new public resource and that added value to the community. A key attribute of this project was the use of reflection, such as collective journaling and exploring art-based tools, in conjunction with children’s direct experiences in the park. This process helped to broaden children’s thinking, interpretations, and communication about their park experiences, while writing the guide.

The SD Forest Exploration Project engaged pre-K and kindergarten children over two academic years in planned educational experiences, including roleplay in imaginary wooded settings, playing and exploring in a small wooded area in a botanic garden, and reflection activities, such as drawing, painting, composition, and journaling about forests. By the end of the first year, a group of four children had written a fictional story about animals saving the forest from a dangerous entity, called “Hombre-árbol” (Man-tree). In the second year, children decided to compose and perform a screenplay for a short film, which was recorded in the botanic garden woods. This was the first movie written by Dominican children, and it was presented at the 6th Dominican Global Film Festival.

In both projects, children had the opportunity of free play. Play is the leading interest and pursuit for 3–6-year-old children (Bredekamp, 2004; Karpov, 2005; Paley, 2009) and adults’ mediation in children’s play promotes children’s mental processes (Bredekamp, 2004; Karpov, 2005). Nature stewardship should be designed as a play-based pedagogy, honoring both children’s free play and adult mediation to promote children’s contribution and reflection.

These and other projects that use direct experiences and reflection to connect children to nature and enable them to contribute to their community represent an investment in young children’s ability to be productive members of society. The children’s accomplishments, including producing a park guide and a film, are the return on investment. Although we did not conduct research on the project outcomes for children, the first author’s observations and the literature would suggest additional returns on investment, including children’s development of socio-emotional, cognitive, and academic skills, connection to nature, and sense of contribution.

## Components of a young children’s stewardship project

Providing the affordances for children to become agents of change requires time and strong ethics. For example, educators should familiarize themselves with the community’s socio-cultural and historical context and develop a trusting relationship and rapport with the children. Additionally, adults must be well equipped to facilitate children’s authentic participation and decision-making and to design adequate educational experiences based on children’s needs and interests. Further, three components—reflection, non-objectification of nature, and a shared strong image of a child—are crucial investments in stewardship programs that yield returns for communities and children.

### Reflection

To guide young children in stewardship will require not only an investment in planning and implementing age-appropriate hands-on activities, but also in designing age-appropriate means for young children to reflect on those activities. Reflection is the process of (re)constructing participation, practice, knowledge, or issue with the aim of impacting the mental schema of an individual, and therefore, promoting behavioral change (Korthagen et al., 2001). Reflection activities can allow children to connect stewardship to broader understanding and an awareness of the importance of their actions. When children share their reflections with adults, adults recognize young children’s perspectives, knowledge, and learning processes, and support them to effect change.

### Non-objectification of nature

Stewardship programs should teach children about nature’s agency and nature as a teacher (Elliott and Davis, 2009; Cutter-Mackenzie-Knowles et al., 2020). Reflection prompts in stewardship programs could relate to this shared agency: How did you care for nature today? How is nature taking care of you today? What did we learn from non-human nature today?

### A shared strong image of the child

According to Malaguzzi (1994), adults have “images of the child” (p. 1) that mediate the way they connect with a child, which in turn impacts the child’s image of the way adults act toward, get to know, hear, and pay attention to a child. Salamon and Harrison (2015) add that early childhood educators’ images of children guide their pedagogies, and therefore, facilitate or limit children’s experiences. The SD projects described above were only possible due to the preschool community’s shared support and ethos about the image of the child as capable, full of potential, and with the right to participate in authentic and joyful learning experiences.

## Final remarks

To what extent do these interventions support the development of contribution, connection to nature, and children’s understanding of their ability to regenerate nature? This is a question to be answered in further research. Researchers might examine children’s learning, the quality of the children-nature interaction, and environmental and other outcomes. Longitudinal or retrospective studies also will promote understanding of the influence of stewardship on children throughout the lifespan.

Young children can be nature stewards *now*. They can be Dr. Seuss’s (1971) Lorax who “speaks for the trees” or the child that received the seed and the message from the Lorax: “UNLESS someone like you Cares a whole awful lot, Nothing is going to get better. It’s not.” Children have agency and the right to participate and should not have to wait until their youth or adulthood to engage in nature conservation and restoration initiatives.

## Author contributions

ED: substantial contributions to the conception or design of the work, or the acquisition, analysis or interpretation of data for the work, and drafting the work or revising it critically for important intellectual content. MK: drafting the work or revising it critically for important intellectual content, provide approval for publication of the content, agreed to be accountable for all aspects of the work in ensuring that questions related to the accuracy or integrity of any part of the work are appropriately investigated and resolved. Both authors contributed to the article and approved the submitted version.

## Footnotes

1 Environmental Education will be used in this paper as a broad construct to refer to children and nature educational experiences. This is the most common term used in North America, although we recognize other terms such as ESD and Education for Sustainable Development are commonly used in other regions with similar albeit slightly different meanings (Elliott and Davis, 2009; Stevenson et al., 2013; Ernst and Burcak, 2019).
